# Personal Aging Is Political: A Feminist Perspective on Subjective Aging

**DOI:** 10.1093/geroni/igaa057.1827

**Published:** 2020-12-16

**Authors:** Anne Barrett, Cherish Michael, Jessica Noblitt

**Affiliations:** Florida State University, Tallahassee, Florida, United States

## Abstract

An extensive literature examines subjective aging – a construct encompassing many aspects of individuals’ views of aging, such as age identity, aging anxiety, awareness of aging, and views of life stages. A factor receiving attention within this research is gender, with studies revealing much about gender differences not only in subjective aging but also its health and behavioral consequences. However, we argue that the literature is limited by its focus on gender as an individual-level characteristic – rather than a profoundly social element emerging within interactions, pervading institutions, and constituting a system of inequality that intersects with others, including age. Addressing this limitation, our chapter applies a feminist perspective to the study of subjective aging. This perspective draws into focus the implications for subjective aging of gender’s social embeddedness and provides an illustration of the interconnection between the personal and political spheres.

